# Preparation and characterization of diatomite and hydroxyapatite reinforced porous polyurethane foam biocomposites

**DOI:** 10.1038/s41598-020-70421-3

**Published:** 2020-08-06

**Authors:** Sibel Demiroglu Mustafov, Fatih Sen, M. Ozgur Seydibeyoglu

**Affiliations:** 1 Nanotechnology and Nanoscience, İzmir Katip Çelebi University, İzmir, Turkey; 2 Sen Research Group, Department of Biochemistry, Faculty of Arts and Science, Dumlupınar University, Evliya Çelebi Campus, 43100 Kütahya, Turkey; 3 Department of Material Science and Engineering, İzmir Katip Çelebi University, İzmir, Turkey

**Keywords:** Biomaterials, Nanoscale materials, Structural materials

## Abstract

Porous three-dimensional (3D) polyurethane-based biocomposites were produced utilizing diatomite and hydroxyapatite as fillers. Diatomite and Hydroxyapatite (HA) were utilized to reinforce the morphological, chemical, mechanical, and thermal properties of polyurethane foam (PUF). Diatomite and Hydroxyapatite were added into polyurethane at variable percentages 0, 1, 2, and 5. The mechanical properties of PUF were analyzed by the compression test. According to the compression test results, the compressive strength of the polyurethane foam is highest in the reinforced foam at 1% by weight hydroxyapatite compared to other reinforced PUFs. Scanning electron microscopy (SEM) images presented structural differences on foam by adding fillers. Functional groups of PUF were defined by Fourier Transform Infrared Spectroscopy (FTIR) and the thermal behavior of PUF was studied with Thermogravimetric Analysis (TGA). The obtained results revealed that PUF/HA biocomposites indicated higher thermal degradation than PUF/Diatomite biocomposites.

## Introduction

The bone can be regarded as a composite substance composed of inorganic minerals mostly formed through hydroxyapatite (HA), which reinforces the bone structure and supplies mechanical strength and moreover an organic matrix consisting of type I collagen. Bone is good at self-regeneration, but the natural healing mechanism is difficult when significant bone loss, so medical implants are generally utilized to overcome the defect of bone. The temporary 3D scaffold, according to the bone tissue engineering approach, plays a significant role in controlling osteoblast functions as well as a fundamental role in guiding the new formation of bone into preferred forms^1^.

Scaffolds for bone regeneration serve primarily as substrates for binding and proliferation of osteogenic cells. Thus, like bone grafts, three-dimensional biodegradable materials with a porous structure were of big interest not only for their structural and composition similarities to the natural bone but also for their original functional properties, like greater surface area, and high mechanical strength^2^. The most studied synthetic biodegradable polymers studied for bone tissue engineering include polylactic acid (PLA), polycaprolactones (PCL), polyglycolide (PGA), and polylactic-co-glycolic acid (PLGA)^3,4^. However, when artificial biodegradable polymers are used, the right equilibrium among the rate of tissue regeneration and in vivo degradation is hard to accomplish. For this reason, other polymers, especially polyurethanes, can be utilized to produce scaffolds for the regeneration of bone tissue. The use of polyurethane for scaffolding is probable to achieve a much wider range of morphological and mechanical characteristics compared to mostly used biodegradable polymers^5^.

Polyurethanes (PU) are classically produced by polyaddition reactions of isocyanates with polyols (polyalcohols). Based on the formulation, a wide property spectrum from elastomer to rigid foams are readily designed and synthesized^6–8^. Currently, they are considered one of the most multipurpose polymeric materials class since they can be used in many types and in a large number of applications including polymers for electronics^9^, adhesives and coatings^10–12^, and biomedical applications^13–15^, etc. Among PU foam types, rigid PUF has enclosed cell structure with low density, poor thermal conductivity, low moisture permeability, superior compression strength, and great strength-to-weight ratio^16–18^. The manufacturing of non-renewable products has been under control in the industry because it causes contamination of the atmosphere. Hence the industry has shifted to more eco-friendly renewable sources and the alternatives produced are the composites from PU and natural sources. Thus, PU biocomposites' main purpose is to decrease the environmental impact of non-organic engineered and petroleum-based fillers^19,20^.

While biodegradable PU is one of the most biocompatible materials employed in scaffolds as temporal extracellular matrices, the major concern about biodegradable polyurethane is absence of bioactive groups that restrict their use^21,22^. An alternative to this issue is to mix PU with bioactive ceramic particles like hydroxyapatite or tricalcium phosphate^22–26^. In this way, the combination of ceramic and polyurethane can increase bioactivity as well as improve the mechanical properties of porous scaffolds^27^. Hydroxyapatite (Ca10(PO4)6(OH)2), a reinforcing particle, is a bioactive and biocompatible substance with a crystal structure found in hard tissues like teeth and bones^28^. HA, which has been shown to stimulate osteoconduction, is widely used in dentistry and orthopedics due to its near biocompatibility with the human body, as well as its strong bone integration. Furthermore, HA has been suggested to be the best alternative for bone, as it offers different conformation possibilities such as the production of powder, coating, and porous bodies^29–31^. HA, which can integrate into bone without causing an immune reaction^32^, is a good material for bone scaffold because it has many advantages, but it is very difficult to shape because it is naturally brittle^33^. This major disadvantage is overcome by mixing HA with other materials to form composites such as metals, polymers, and others.

Diatomite, also named diatomaceous earth, consists of fossilized remains of diatoms, that are single-celled water plants having silica cell walls. Lightweight and high purity diatoms, due to their several well-arranged microscopic pores, have unmatched physical properties, like high permeability^34–36^. Diatomite is used as reinforcing fillers, filtering agents, abrasives, medicines, and insulating materials due to its low price and high abundance^37–40^. The unusual three-dimensional biosilica structure of diatoms is an ideal candidate for use as multifunctional scaffolds having free hydroxyl groups over the great frustule layer, making it simple to function with chemical or biological parts^41,42^. In engineering and biomedical applications, some studies have regarded diatom frustules as natural silica particles. The effect on the osteoblast-like SaOs-2 cell line of silicon-replaced hydroxyapatite coatings was investigated employing diatomaceous earth as the source of silicon. In vitro works have shown that Si-Hap coating considerably increases the proliferation and activity of osteoblasts compared to Si-HAp synthetic silica coating^43^. The use of diatom as silicon donor materials in bone tissue engineering areas was studied in another study and the results showed that purified diatom-based microparticles and nanoparticles had restricted or no cytotoxic effect^44^.

The goal of this work is to develop and to analyze polyurethane-based biocomposites using the biomaterial properties of both diatomite and hydroxyapatite. For this reason, the effects of the basic chemistry reactions formed from various combinations of fillers were studied. FTIR was employed to identify functional groups of the polymers. The mechanical and thermal properties were examined using compression test measurements and thermogravimetric analysis. Our research studies focus on the effects of diatomite and hydroxyapatite on morphological, thermal, and mechanical properties while obtaining reinforced porous biocomposite.

## Experimental section

### Materials

Polyether polyol (Elastapor H2011/4) and polymeric methylene diisocyanate (IsoPMDI 92,140) were obtained from the BASF firm. In this study, the ratio of used polyol-PMDI was 100/102. The values of the polyol and isocyanate density are 1.13 g cm^−3^ and 1.23 g cm^−3^, respectively. Nano-sized HA powder was provided by Sigma Aldrich, UK (˂ 200 nm). Diatomite was obtained from local fields near the Aegean region, Turkey.

### Polyurethane foam composite production

The polyurethane foam composite was fabricated in a two-step process. In the first step, 0, 1, 2, and 5% of hydroxyapatite and diatomite were added separately to the polyol to compare the bare polyurethane foam. These solutions were mixed by magnetic stirrer (IKA C-MAG HS 7) for 15 min until homogenized. Since hydroxyapatite is a nanostructured material, sonication (Hielscher Ultrasonics) was used for 15 min for better dispersion after magnetic stirrer. In the second step, isocyanate was added into the mixtures (polyol, polyol/hydroxyapatite, and polyol/diatomite) for getting a reaction. The mixture was mixed at 600 rpm for 20 s by mechanical mixer (Velp Scientifica, Overhead Stirrer) and left to foamed at room temperature. After the foam had hardened for about 45 min at room temperature, the foam was cut to specific dimensions for characterization analyzes.

### Characterization of Polyurethane foam composites

#### Scanning electron microscope (SEM)

The morphology of PUF, PUF/diatomite, and PUF/hydroxyapatite composites were analyzed using scanning electron microscopy (SEM, FEI Qanta Feg 250) with gold-coated samples.

#### Fourier transform infrared spectroscopy (FTIR)

Structural characterization of two different reinforced PUF composites and base PUF were studied with Thermo Scientific, model Nicoletta IS5-ATR mode. The FTIR analysis was completed in the spectral range of 400–4,000 cm^−1^ with absorbance mode. Each spectrum was taken for 64 scans with a resolution of 4 cm^−1^.

#### Thermogravimetric analysis (TGA)

Thermogravimetric analysis was utilized to detect the thermal stability and decomposition temperature for each PUF sample. TGA analysis was measured in the temperature from 30 to 950 °C by Perkin Elmer Diamond TG/DTA. The measurements were done with a heating rate of 10 °C min^−1^ under N_2_ atmosphere. About 5 mg of samples on a platinum pan was used for each analysis.

#### Compression test

The compression tests were done by using a Shimadzu AGS-X universal testing machine with a 5 kN load cell. The compression test of PUF samples was performed according to BS EN ISO 844:2009 test standard by using 50 mm cube samples. The test speed was set to 5 mm/min at room temperature and all values given are means of five measurements.

## Results and discussions

### SEM analysis

**Figure 1:**
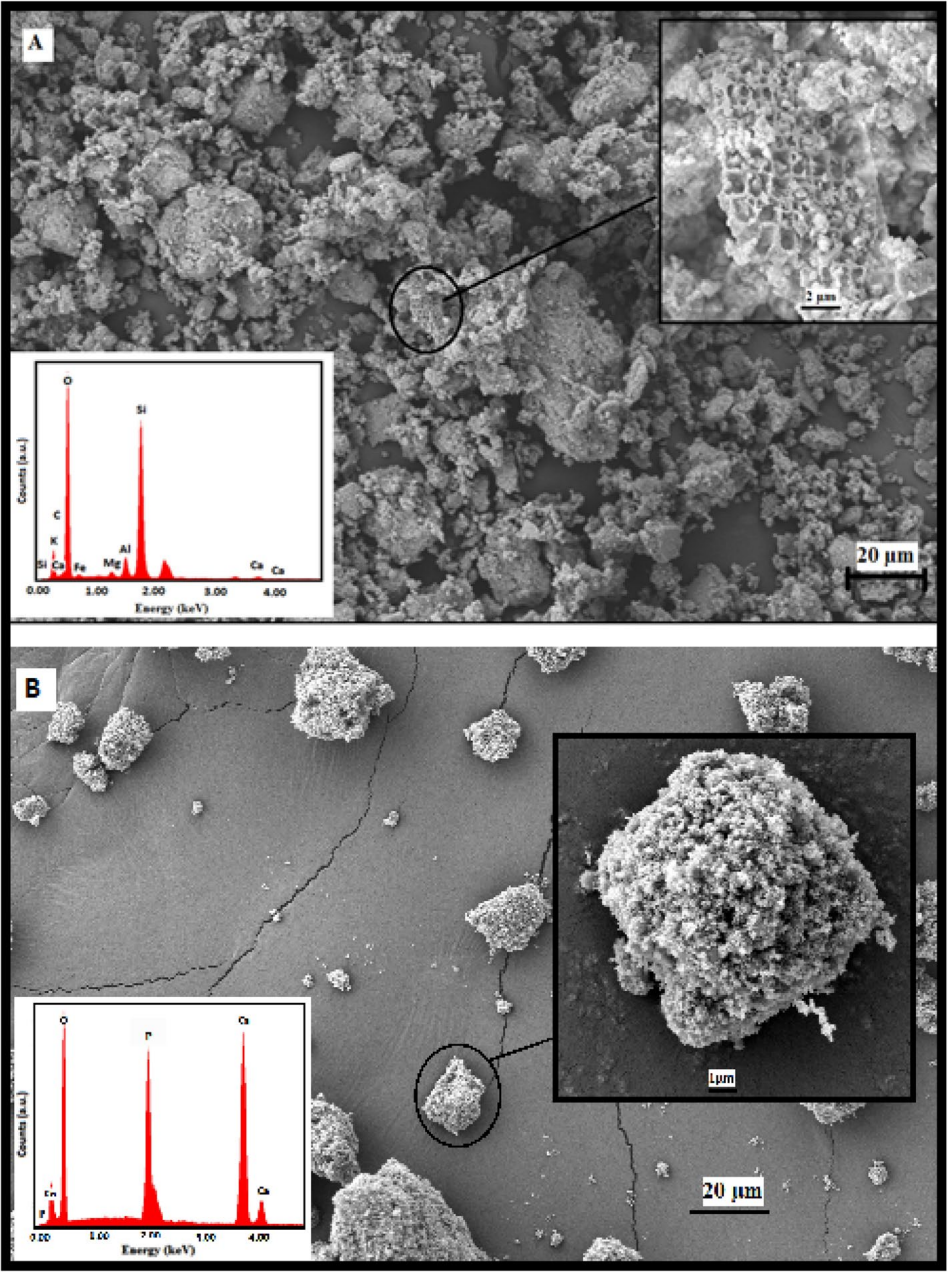
SEM images of (**A**) diatomite and (**B**) hydroxyapatite, respectively [insets: higher magnification SEM image (top), EDX spectrum (bottom)].

Figure 1a indicates the surface morphology by SEM of the diatomite and SEM images show that the diatomite exhibits different morphology and highly porous structure (top inset). All diatomite particles exhibit a hierarchical porosity ranging in size from µm to nm. The EDS spectra presented the existence of Si and O, being both powders basically composed of silica (bottom inset, Fig. 1a). Naturally gathered silica normally does not contain toxic heavy elements and its porous structure is given a possibility for bone cell adhesion. Figure 1b indicates the SEM images of the hydroxyapatite and the hydroxyapatite particles formed were immensely agglomerated. This may be due to the particle-by-particle interaction of hydroxyapatite and they begin to agglomerate. Also, from the spectrum of EDX as shown in Fig. 1b (bottom inset), the typical Ca, P, and O peaks belonged to hydroxyapatite are available. On account of its structural and chemical similarity to the mineral phase of bone, hydroxyapatite is generally utilized in hard tissue repair.

**Figure 2:**
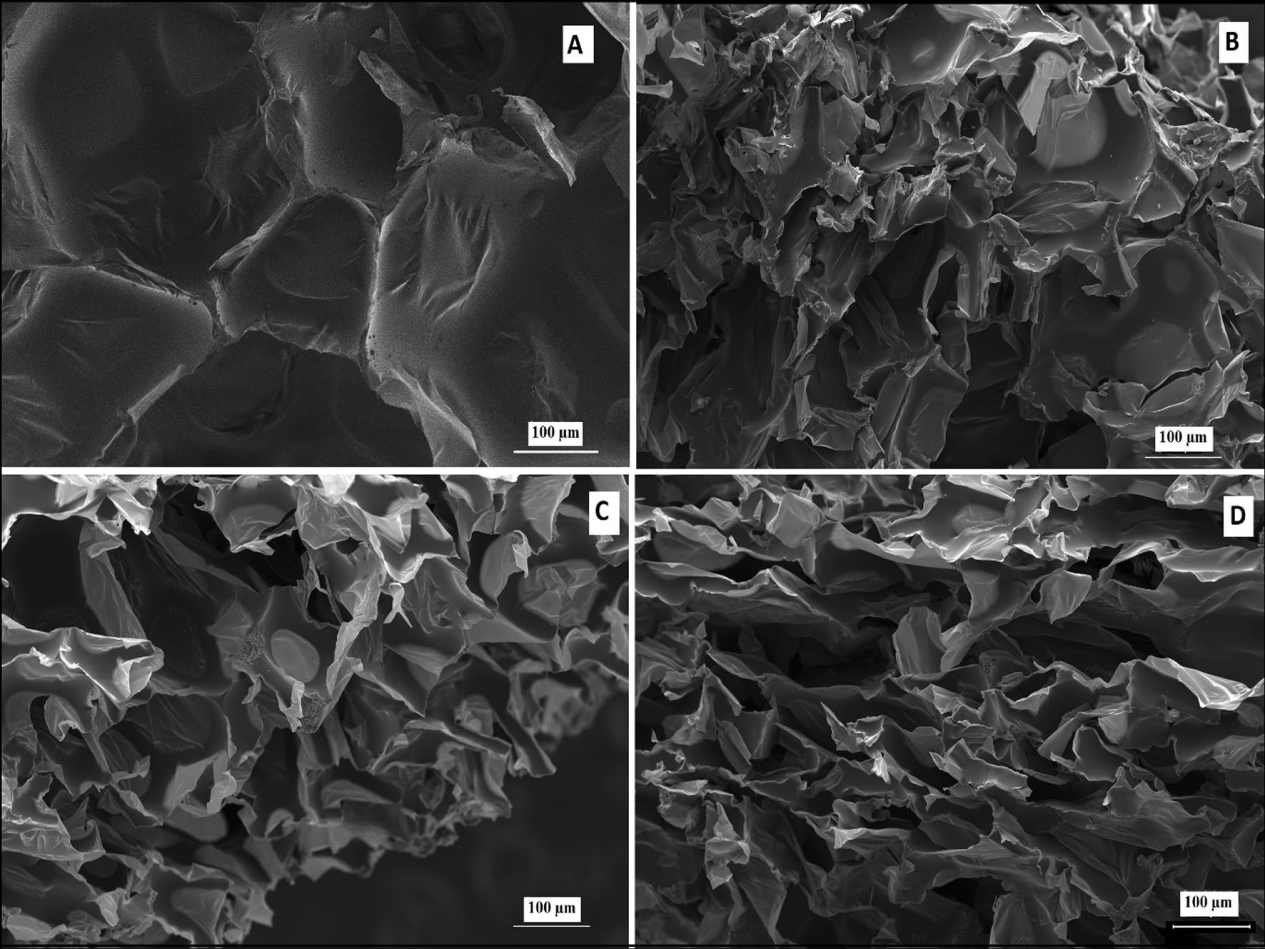
SEM images of the PUF reinforced with (**a**) 0% diatomite; (**b**) 1% diatomite; (**c**) 2% diatomite and (**d**) 5% diatomite.

Figure 2 indicates the SEM images of pure and PUF/diatomite biocomposites with diatomite content at varying 1%, 2%, and 5%. From SEM images, it is seen that PUF consists of the distribution of microporous cells in irregular polyhedral form. However, when diatomite was added as a supporting material, it was observed that the PUF biocomposites had a rougher surface and pore diameters smaller than PUF. The reduced cell size may be owing to the silica acting as a nucleating agent, and the deformation of the cell walls is increased with increasing filler concentrations^45,46^.

**Figure 3:**
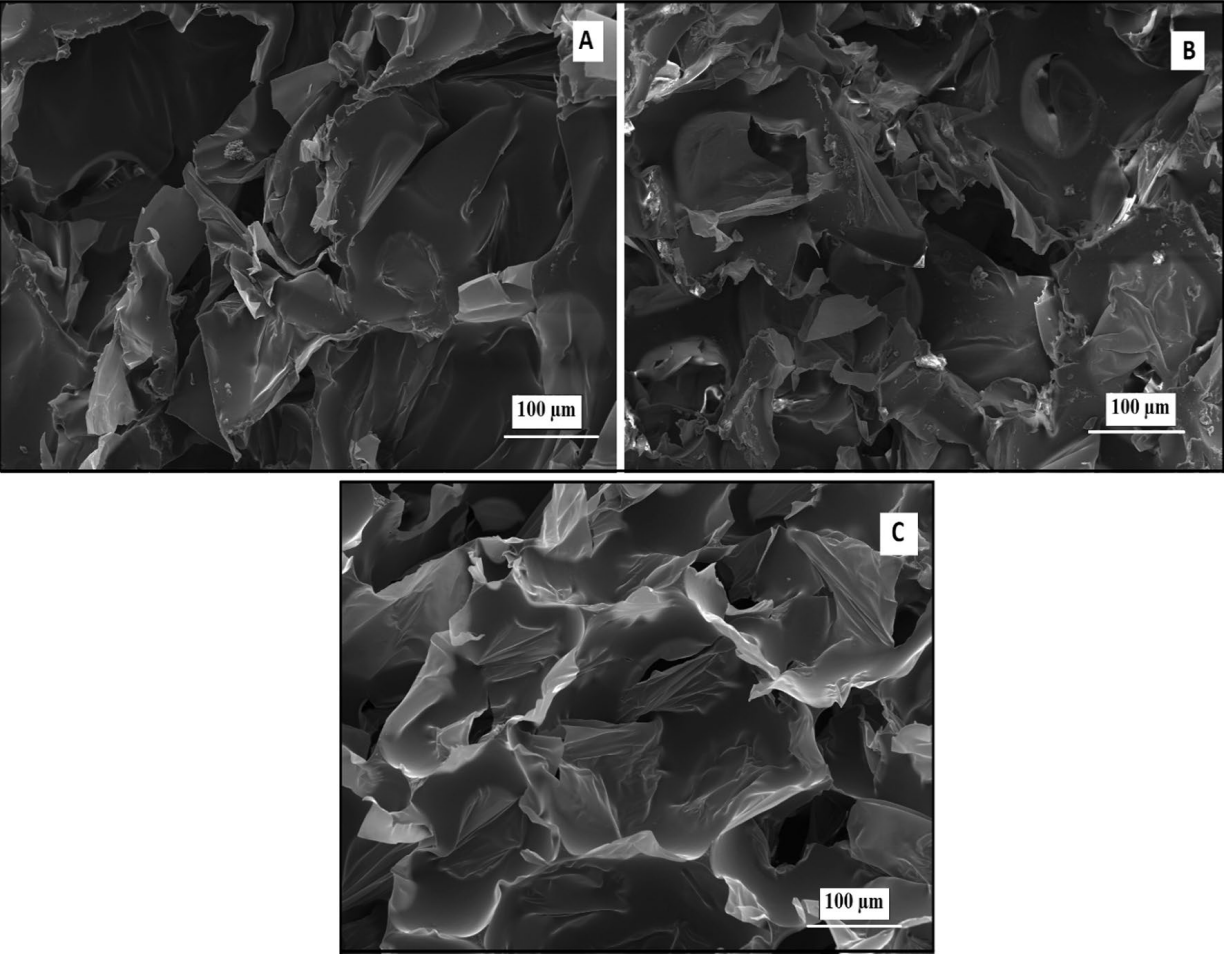
SEM images of the PUF reinforced with (**a**) 1% HA; (**b**) 2% HA and (**c**) 5% HA.

SEM images of porous polyurethane foams for three different HA concentrations are indicated in Fig. 3. From SEM images of Fig. 3, it is clear that PUF/HA biocomposites indicated heterogeneous 3D porous structure with polyangular pores, but it was observed that hydroxyapatite particles were adhered to pores walls because of without a completely homogenous distribution. The sonicator time may be increased to solve the problem of homogeneous distribution of HA in the PUF. In addition, the surface of these porous bio-composites is rough and this can help to promote cellular adhesion and induce new bone formation^47,48^.

### FTIR analysis

In order to observe the distinctions in bond structure, PUF, Diatomite/PUF, and HA/PUF samples were subjected to FTIR analysis. The FTIR spectra of the produced PUF sample is presented in Figure S1. The specific peak around 3320 cm^−1^ was attributed to the hydrogen-bonded NH groups, and the sharp peak around 2875 cm^−1^ was corresponding to asymmetric and symmetric –CH_2_ groups. For the synthesis of polyurethane foam, the FTIR peak at 2271 cm^−1^ is quite significant because it corresponds to the free, unreacted isocyanate peak in PU structure^49^. Figure S1 shows that peak is seen at 2271 cm^−1^ and thus it is understood that the PUF sample still contains some unreacted isocyanate monomers. However, Jiao et al. showed that this peak can disappear with room temperature rising to 280 °C in air and 320 °C in nitrogen^50^. In addition, the peak in the range 1709–1715 cm^−1^ was related to PUF's free and hydrogen-bonded –C=O groups. Also, the strong peak at 1509 cm^−1^ was related to stretching vibrations of the –NH and the peak around at 1220 cm^−1^ was corresponding to the vibration of C–O–C groups^51,52^.

The FTIR spectra of diatomite and the reinforced PUFs with different diatomite content biocomposites are presented in Figure S2. The FTIR spectrums of diatomite display major peaks at 3647, 1629, 1488, 1116, and 791 cm^−1^, as represented in Figure S2. The peak at 3647 cm^−1^ is owing to the stretching vibrations of the internal OH groups and the peak at 1629 cm^−1^ indicates H–O-H bending vibration of adsorbed water in diatomite. The peak at 1488 cm^−1^ is owing to the C-H_2_ deformation^53,54^. The peak at 1116 cm^−1^ can be attributed to Si–O–Si stretching and the peak at 791 cm^−1^ represents SiO–H vibration^55,56^. Obviously, all these characteristic peaks can be found in the FTIR spectrum of Diatomite/PUF samples and the intensity of these peaks enhanced with diatomite content in biocomposites. However, the peak intensities of PUF biocomposites containing 2% and 5% diatomite are similar.

The FTIR spectrum of HA and the combined spectra of PUF/HA biocomposites with various HA content are presented in Figure S3. The absorption peaks around 2922–2930 cm^−1^ represent the characteristic peaks of methyl and methylene groups while the peak at 1475 cm^−1^ is related to carbonic group (CO_3_^−2^) in HA. The intensity of these peaks increased with increasing in hydroxyapatite content in PUF/HA biocomposites. For the PO_4_^3−^ group, an absorption peak at 1023 cm^−1^ was displayed. However, the disappearance of this peak in the spectrum of PUF/HA biocomposites can be attributed to a chemical bond formed between the polyurethane and HA^57,58^. In addition, XRD analysis was done to better understand the chemical composition of the sample, and the results of the analysis are provided in the Supporting Information (Figure S4). According to the data from this analysis, the specific crystalline peaks of hydroxyapatite at 2θ = 25.87°, 28.98°, 29.02°, 31.71°, 33.87°, 39.81°, 46.59°,49.37°, and 63.87° can be assigned to (002), (102), (210), (211), (300), (310), (222), (213), and (304)^59,60^. The XRD pattern of diatomite displays SiO_2_ features centered at 2θ = 21.93°^61,62^. Also, quartz impurity was seen at 2θ = 26.64° in the diatomite sample (Figure S4)^63^.

### TGA analysis

TGA was done to analyze the behavior of thermal decomposition and understand the diatomite and hydroxyapatite effect on the thermal stability of the polyurethane foams.

**Figure 4:**
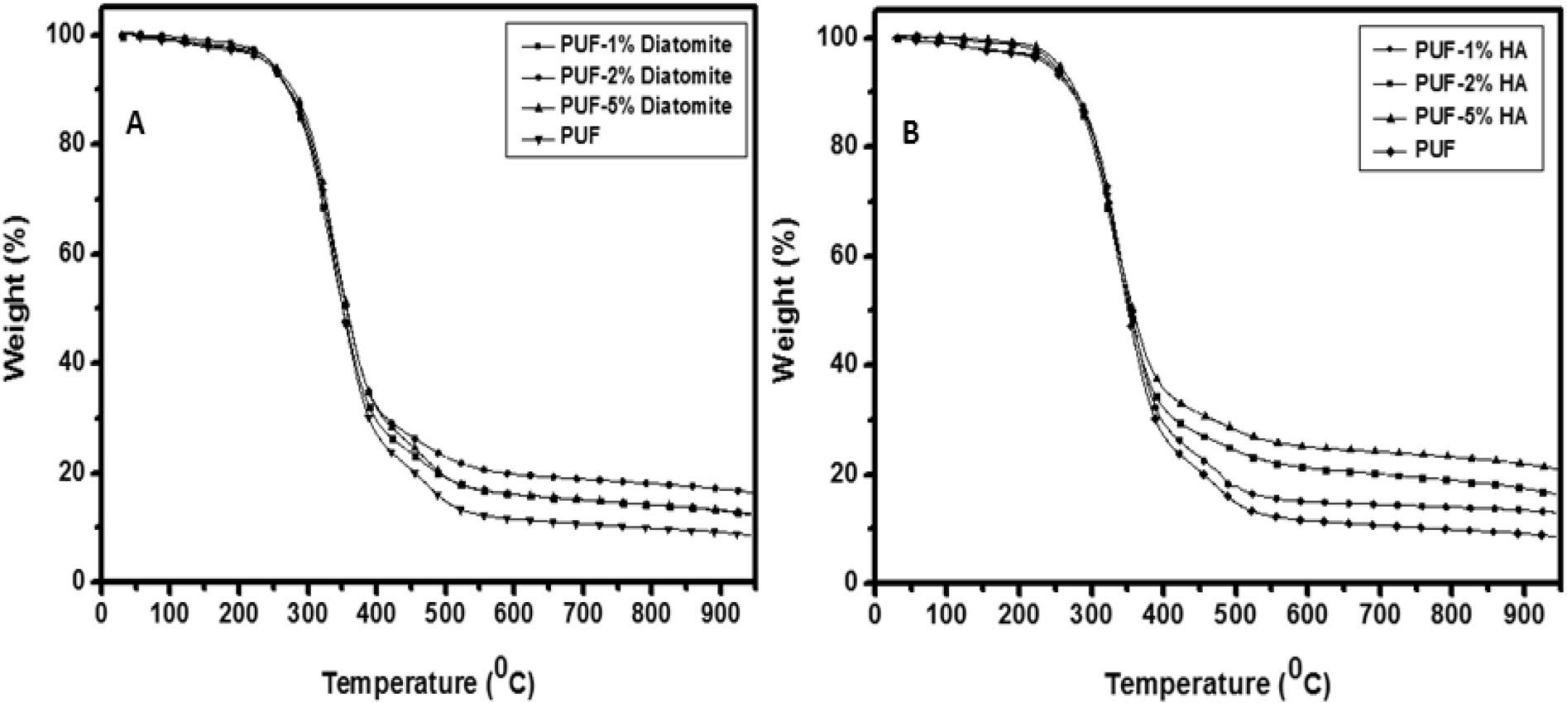
(**a**) TGA curves of the PUF-Diatomite biocomposites with pure PUF and (**b**) TGA curves of the PUF-HA biocomposites with pure PUF.

In Fig. 4a, the thermal behavior of reinforced PUFs with different diatomite content is compared to pure PUF. The TGA results show that PUF samples indicate similar behaviors and the first weight loss is observed at ~ 40 °C, because of the evaporation of water from the PUF samples. Similarly, in the literature, it has been shown that the first step in the thermal decomposition process of diatomites occurs among 25 °C and 100 °C is related to absorbed water or mechanically compressed^64^.

As can be seen from the onset values shown in Table S1, it is seen that the second weight loss takes place among 279 °C and 399 °C with respect to thermal degradation of the polymer structure. The presence of diatomite was observed to cause a reduction in the initial decomposition temperature of the biocomposites, but it was found to cause improvement at the temperature at which the degradation ended (Table S1). In particular, biocomposite PUFs containing 2% and 5% diatomite are resistant to higher temperatures than pure PUF. Furthermore, as indicated in Fig. 4a, the total weight loss of the biocomposite PUFs measured in the TGA analysis is low in PUF containing 2% diatomite; this indicates that more than 2% diatomite additive does not significantly affect the thermal stability of the biocomposites.

The curves of thermogravimetric analysis for thermal degradation of reinforced PUFs with pure PUF and hydroxyapatite are displayed in Fig. 4b. The initial weight loss for HA reinforced PUFs occurs at ~ 35 °C and approximately 1% weight loss is because of evaporation of the remaining water in the PUF samples. Biocomposite PUFs containing hydroxyapatite exhibit slow weight loss between 279 and 396 °C (Table S1). In the light of the data obtained from the TGA analysis results shown in Fig. 4b, the sample with the lowest total weight loss is PUF containing 5% HA. TGA results noticeably present that the higher the amount of HA in biocomposites, the higher the thermal stability^23^.

### Compression testing

**Figure 5:**
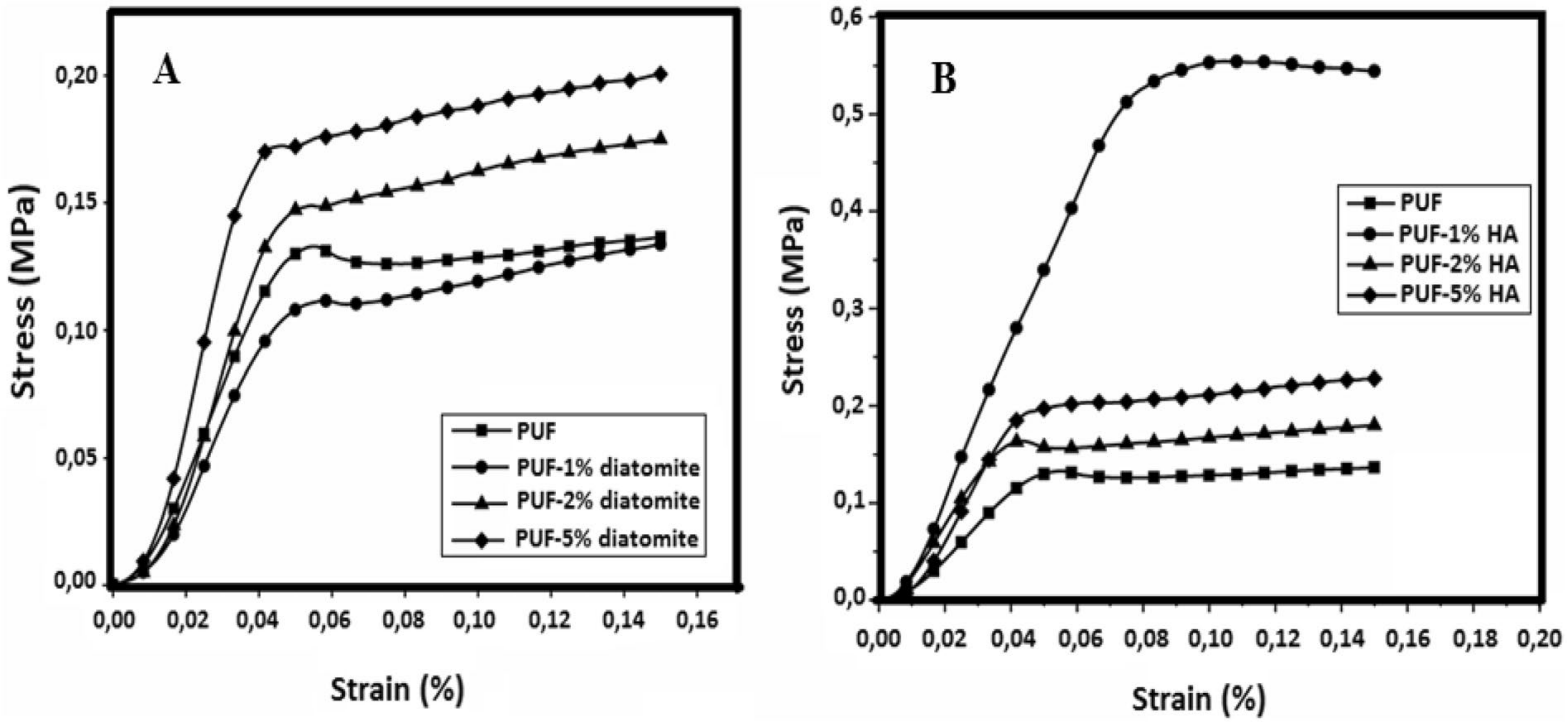
Stress–strain curves for (**a**) PUF and PUF/Diatomite biocomposites and (**b**) PUF and PUF/HA biocomposites.

Polymer matrix reinforcements are typically used to improve mechanical strength and structure modulus. This enhanced reinforcement effect in the polymer matrix depends on particle shape, size, and dispersion. The effects of diatomite and hydroxyapatite have been studied on the mechanical properties of PUFs. The compression stress–strain curves of PUF, PUF reinforced with diatomite, and PUF reinforced with HA are shown in Fig. 5. As can be seen from the curves, there is an increase due to the increase in concentration relative to pure PUF when it exceeds 1% by weight of diatomite^65^.

Compared to other biocomposites, a significant increase in 1% HA was observed and the increase was expected to continue after 1 percent due to its compact nature^23^, but the desired increase could not be achieved. It is assumed that when the nanostructure incorporated more than 1 percent of PUF, total homogeneity in the mixture was not achieved. Another reason is its agglomeration, which can inhibit the homogeneous distribution of HA in PUF, as shown in Fig. 1b.

Compressive modulus and max compressive strength of pure PUF, diatomite-filled PUF and hydroxyapatite were measured as a result of the compression test (Table 1). The compressive strength is regarded as the max stress in the stress–strain curve, and the compression modulus is determined in the elastic region as presented in Fig. 5 from the slope of the stress–strain curve. Pure PUF's compressive strength is 137.29 kPa and the compressive modulus is 3.58 MPa. Max values (7.45 MPa and 553.61 kPa) were obtained with the addition of 1 weight percent HA in both properties.

**Table 1:** Compressive modulus and maximum compressive strength values of diatomite and hydroxyapatite reinforced PUF.

Filler concentration (%)	Diatomite		Hydroxyapatite	
	Compressive modulus (MPa)	Max compressive strength (kPa)	Compressive modulus (MPa)	Max compressive strength (kPa)
0	3.58	137.29	3.58	137.29
1	3.45	133.71	7.45	553.61
2	4.18	174.93	5.45	179.97
5	6.56	200.56	6.31	228.18

The compressive properties of PUF reinforced with diatomite are lower than those of PUF reinforced with HA. Therefore, it is considered that the compression properties of the PUF can be improved by providing more than 1% by weight but more homogeneity with HA.

## Conclusion

Hydroxyapatite and diatomite with their unique properties were demonstrated in this study as proper candidates to strengthen polyurethane foam in terms of morphological, thermal, and mechanical properties. SEM, FTIR, TGA, and mechanical analyses were done to understand the effects of hydroxyapatite and diatomite reinforcing on chemical and physical properties of polyurethane-based biocomposites. Data of the porous biocomposite based on PUF fabricated in this study indicated that diatomite enhanced the surface area and roughness of PUF and also hydroxyapatite improved its thermal stability and mechanical strength. Although the developed biocomposites do not have sufficient compressive strength similar to compact human bone, their exceedingly porous structures provide increased surface area that facilitates cell attachment.

## Supplementary information

Supplementary file 1
