# Supplementary material for: Preparation and characterization of diatomite and hydroxyapatite reinforced porous polyurethane foam biocomposites
Source: Sci Rep. 2020 Aug 6;10:13308. doi: 10.1038/s41598-020-70421-3 (PMC7413266; doi:10.1038/s41598-020-70421-3)
Supplement: Supplementary file 1 [file 41598_2020_70421_MOESM1_ESM.docx]

**SUPPORTING INFORMATION**

**Preparation and Characterization of Diatomite and Hydroxyapatite Reinforced Porous Polyurethane Foam Biocomposites**

Sibel Demiroglu Mustafov^1^, Fatih Sen*^2^, M. Ozgur Seydibeyoglu*^3^

^1^Nanotechnology and Nanoscience, İzmir Katip Çelebi University, İzmir, Turkey

^2^Sen Research Group, Department of Biochemistry, Faculty of Arts and Science, Dumlupınar

University, Evliya Çelebi Campus, 43100 Kütahya, Turkey

^3^Department of Material Science and Engineering, İzmir Katip Çelebi University, İzmir, Turkey

*Corresponding authors: seydibey@gmail.com, fatih.sen@dpu.edu.tr

Tel: +90(232) 329 35 35; +90 (274) 265 20 31

**
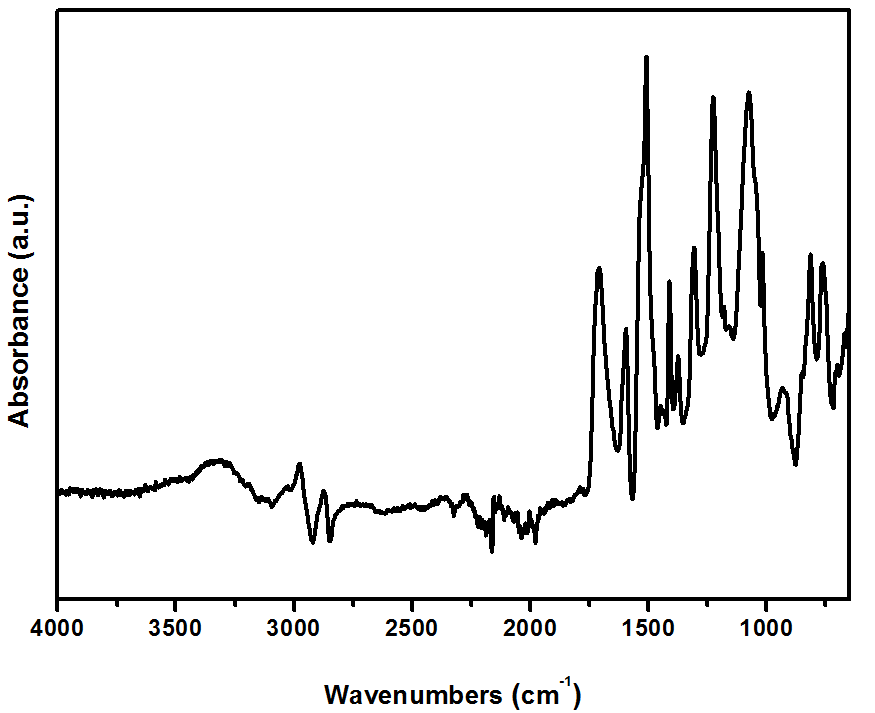
**

**Figure S1.** FTIR spectrum of PUF


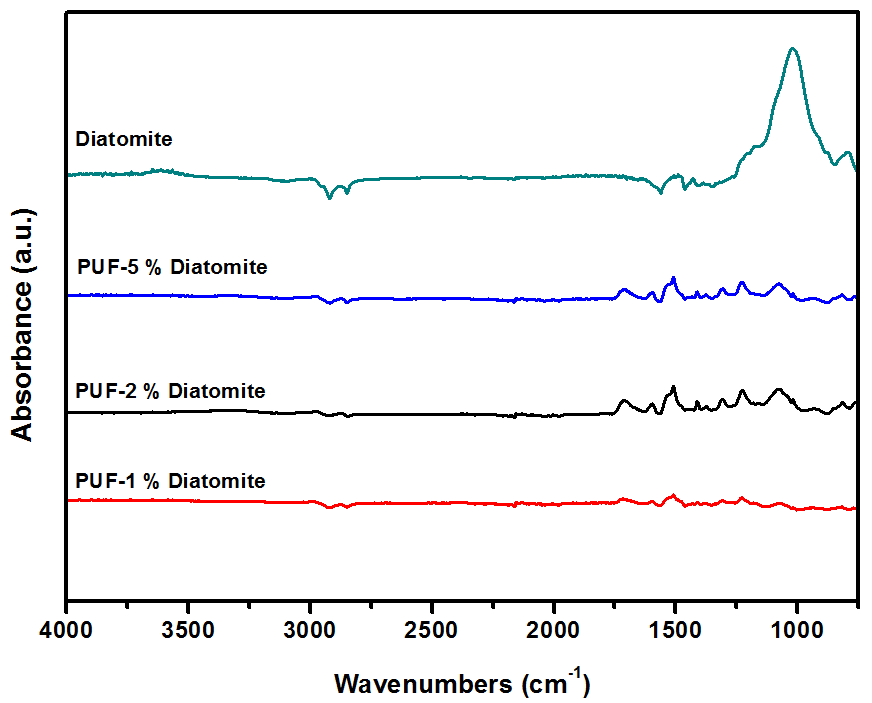


**Figure S2:** FTIR spectrum of diatomite and the reinforced PUFs with different diatomite content


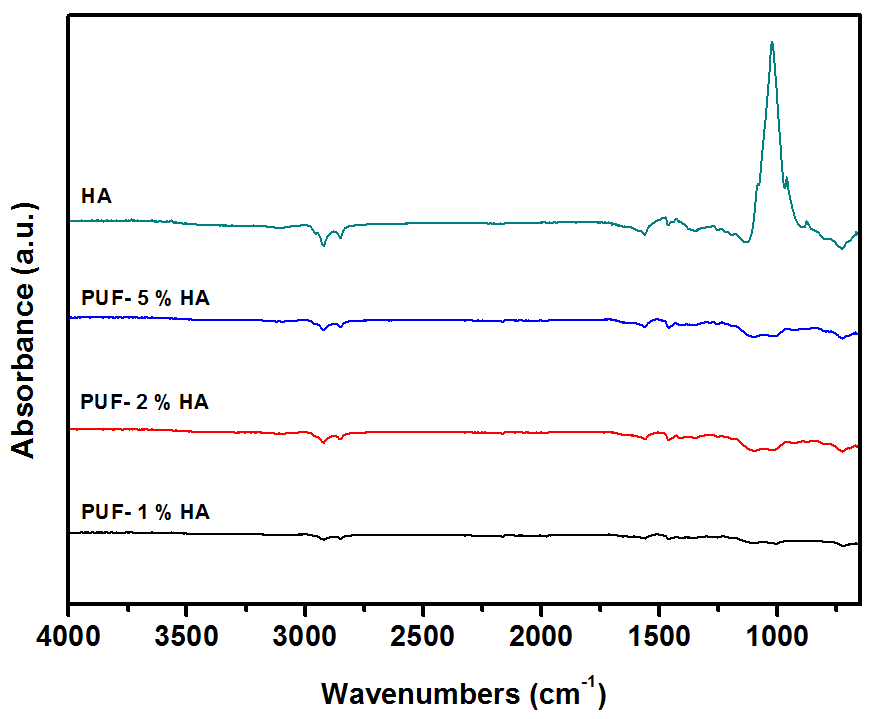


**Figure S3:** FTIR spectrum of HA and the reinforced PUFs with different HA content


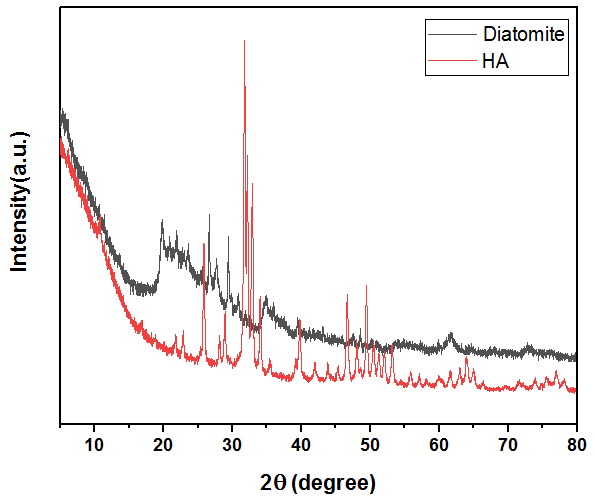


**Figure S4:** XRD pattern of diatomite and hydroxyapatite

**Table S1:** TGA parameters for the PUF, PUF-Diatomite biocomposites, and PUF-HA biocomposites

|  | Onset | End | Step Inflection Point |
| --- | --- | --- | --- |
| PUF | 290.72 °C | 394.46 °C | 335.71 °C |
| PUF-1% Diatomite | 280.82 °C | 391.52 °C | 329.88 °C |
| PUF-2% Diatomite | 279.66 °C | 398.67 °C | 339.16 °C |
| PUF-5% Diatomite | 287.74 °C | 399.83 °C | 331.14 °C |
| PUF-1% HA | 290.88 °C | 396.67 °C | 335.69 °C |
| PUF-2% HA | 279.01 °C | 389.92 °C | 331.38 °C |
| PUF-5% HA | 279.41 °C | 386.31 °C | 333.48 °C |
